# Overlapping and divergent signaling pathways for ARK1 and AGD1 in the control of root hair polarity in *Arabidopsis thaliana*

**DOI:** 10.3389/fpls.2013.00528

**Published:** 2013-12-24

**Authors:** Cheol-Min Yoo, Elison B. Blancaflor

**Affiliations:** Plant Biology Division, The Samuel Roberts Noble FoundationArdmore, OK, USA

**Keywords:** Arabidopsis, ARF-GAP, cytoskeleton, kinesin, membrane trafficking, root hairs, tip growth

## Abstract

We previously showed that seedlings harboring mutations in genes encoding ARK1, an armadillo repeat-containing kinesin, or AGD1, a class 1 ARF-GAP, have root hairs that exhibit wavy/spiral growth and two tips originating from one initiation site. These root hair defects were accompanied by bundling of endoplasmic microtubules and filamentous actin (F-actin) that extended to the extreme root hair apex. The similar phenotypes of *ark1* and *agd1* mutants suggest a tight coordination between the cytoskeleton and membrane trafficking in the control of root hair polarity. Indeed, cell biological and genetic studies of the *agd1* mutant provided evidence that AGD1's involvement in root hair development involves cross-talk among phosphoinositides (PIs), the actin cytoskeleton and other small GTPases such as ROP2 and RABA4b. Here we show that *ark1* root hairs mirror those of *agd1* with regard to altered targeting of ROP2 and RABA4b, as well as abnormal tonoplast organization. Furthermore, like *agd1*, enhanced root hair defects in double mutants in *ARK1* and genes encoding a type B phosphatidylinositol-4-phosphate 5-kinase 3 (*PIP5K3)*, a phosphatidylinositol-4-phosphate (*PI-4P*) phosphatase (*RHD4*), a phosphatidylinositol transfer protein (*COW1*), and a vegetative actin isoform (*ACT2*), were observed. However, root hair shape of some *ark1* double mutant combinations, particularly those with *act2, pip5k3* and *rhd4* (*ark1 act2*, *ark1 pip5k3*, *ark1 rhd4*), differed in some respects from *agd1 act2, agd1 pip5k3*, and *agd1 rhd4*. Taken together our results continue to point to commonalities between ARK1 and AGD1 in specifying root hair polarity, but that these two modulators of tip-growth can also regulate root hair development through divergent signaling routes with AGD1 acting predominantly during root hair initiation and ARK1 functioning primarily in sustained tip growth.

## Introduction

Root hairs are long, tubular extensions of specialized root epidermal cells called trichoblasts. Their formation is typically triggered by the pH-dependent loosening of the trichoblast cell wall, which is visually manifested as slight bulging at a specific site on the trichoblast surface (Bibikova et al., 1998). This so called “initiation stage” is followed by a period of cell elongation where growth is confined to the extreme tip of the root hair cell, a process known as tip growth. As a result, a fully expanded root hair cell assumes the shape of a straight tube with a consistent diameter. The highly predictable growth of a root hair has made it a good model system to identify molecular components of polarity establishment in plant cells (Rounds and Bezanilla, 2013).

An essential component of the root hair growth machinery is the trafficking of vesicles containing cell wall and membrane precursors that must be directed to the very tip of the cell to sustain growth. This process is known to be mediated by the actin cytoskeleton, actin binding proteins, calcium gradients and small GTP binding proteins (small GTPases) (Pei et al., 2012; Gu and Nielsen, 2013). Also pivotal for root hair development are the phosphoinositide (PI) group of signaling lipids, which together with their respective metabolic enzymes could function as site-specific signals on the cell membrane that direct elements of the cytoskeleton and the vesicle trafficking complex, such as the exocyst, to defined regions of the cell to maintain tip growth (Heilmann, 2009; Žárský et al., 2009).

Through our previous forward genetic work in *Arabidopsis*, we identified an Armadillo Repeat-containing Kinesin 1 (ARK1) and an Adenosine Diphosphate Ribosylation Factor (ARF)-GTPase Activating Protein (GAP) Domain-containing protein (AGD1) as additional components that specify root hair polarity. Both the *ark1* and *agd1* mutants exhibited wavy and bifurcated root hair growth instead of the straight growth, single growth point phenotype typical of wild-type root hairs (Yoo et al., 2008). ARK1 together with its homologs, ARK2 and ARK3, belongs to a plant specific group of kinesin microtubule motor proteins due to its C-terminal armadillo repeat-containing domain (Richardson et al., 2006). Consistent with its predicted function, the N-terminal kinesin motor domain of ARK1 was shown to bind polymerized microtubules *in vitro* and a green fluorescent protein (GFP)-ARK1 fusion decorated microtubules in transient expression studies (Yang et al., 2007; Yoo et al., 2008). The C-terminal armadillo repeat-containing domain of ARK1 was also demonstrated to bind polymerized actin *in vitro*, leading to the proposal that it coordinates microtubule and F-actin cross-talk during root hair growth (Yang et al., 2007). AGD1 on the other hand is an ARF-GAP, a protein that modulates the activity of the ARF family of small GTPases, which are known regulators of membrane and organelle trafficking. The activity of ARF-GTPases, like other small GTPases, is regulated through a cycle of GTP binding and hydrolysis, which activate and inactivate the ARF-GTPase, respectively. The latter process is mediated by the action of ARF-GAPs (Donaldson and Jackson, 2011). In *Arabidopsis*, there are 15 AGD proteins divided into four classes with AGD1 belonging to the multi-domain class1 ARF-GAPs (Vernoud et al., 2003). AGD1 was shown to localize to punctate bodies reminiscent of the endomembrane system, which support its predicted role as a modulator of vesicle trafficking (Yoo et al., 2008). Recently, we showed that AGD1 impacts root hair polarity by maintaining the correct targeting of various root hair tip growth including Rho Of Plants2 (ROP2) and RabA4B small GTPases, calcium gradients, and PI-4P domains (Yoo et al., 2012).

The *ark1* and *agd1* mutants exhibited disrupted root hair microtubules and F-actin (Yang et al., 2007; Sakai et al., 2008; Yoo et al., 2008). This together with the fact that root hairs of *agd1* and *ark1* resembled wild-type root hairs treated with actin and microtubule inhibitors (Bibikova et al., 1999), has led us to suggest that ARK1 and AGD1 might have overlapping signaling functions in specifying cytoskeletal organization during root hair tip growth (Yoo et al., 2008). However, the observation that low concentrations of brefeldin A (BFA), a fungal macrolide inhibitor of ARF-GTPase activation, causes *agd1*, but not *ark1* root hairs to revert to straight growth, have also pointed to the possibility that AGD1 and ARK1 modulate root hair development through distinct molecular pathways (Yoo et al., 2008).

To further clarify the functional relationship between ARK1 and AGD1 in the control of tip growth, we generated double mutants in *ARK1* and other genes known to affect root hair development (e.g., *ACT2*, *COW1*, *PIP5K3*, and *RHD4*). The resulting double mutants were compared to corresponding double mutants of *agd1* described previously (Yoo et al., 2012). In addition, dynamic imaging of the small GTPases, RABA4b, and ROP2, in root hairs of *ark1* was conducted and compared to *agd1*. Here, we show that like AGD1, ARK1 is involved in maintaining the stability of small GTPases that direct root hair tip growth. However, subtle differences in root hair shape between double mutant combinations to *ark1* and *agd1* continue to point to divergent signaling pathways by which ARK1 and AGD1 mediate polar root hair growth.

## Materials and methods

### Generation of double mutants

All of the *Arabidopsis* lines used in this study are of the Col-0 ecotype. We used *ark1-1* (Salk_035063, a T-DNA mutant of the *At3g56870* gene; Yoo et al., 2008), *agd1-1* (a deletion mutant of the *At5g61980* gene; (Yoo et al., 2008, 2012), *act2-3* (Salk_048987, a T-DNA mutant of the *At3g18780* gene; Nishimura et al., 2003), *cow1* (Salk_002124, a T-DNA mutant of the *At4g34580* gene; Yoo et al., 2012), *pip5k3-4* (Salk_026683, a T-DNA mutant of the *At2g26420* gene; Stenzel et al., 2008), and *rhd4-1* (a point mutant of the *At3g51460* gene; Thole et al., 2008). The *agd1-1* mutant was isolated from a forward genetic screen and described previously (Yoo et al., 2008, 2012) while other single mutants were obtained from the Arabidopsis Biological Resource Center (ABRC). Double mutants were identified by polymerase chain reaction (PCR)-based genotyping or DNA sequencing.

### Growth conditions and evaluation of root hair phenotypes

Seeds of wild-type and mutants were surface sterilized and planted in a half strength of Murashige and Skoog (MS) media containing 0.5% agar layered on 48 × 64 mm coverslips, as described in Dyachok et al. (2009). To analyze root hair phenotypes, 4-day-old seedlings were examined with Nikon Eclipse TE300 stereo-microscope equipped with a 10× Plan Fluor DLL objective and photographed with a Nikon DXM1200 camera (Nikon Corporation, Melville, NY, USA). For measurement of trichoblast length, root tissues were stained with 10 μ M of propidium iodide and examined using the 20× Plan Fluor objective of a Nikon Optiphot-2 microscope equipped with epifluorescence. Images of propidium iodide-stained roots were captured with a Nikon DS-Ri1 camera. Root hair and trichoblast length from the digital images were measured using ImageJ ver. 1.46r software (http://rsbweb.nih.gov). The data were analyzed by One-way Analysis of Variance (ANOVA) to test statistical significance, and Tukey's honestly significant difference (HSD) test for multiple comparisons of means. Statistical analysis was conducted using SPSS ver. 19 software (IBM). For 3D rendered images of double mutant root hairs, root tissues were stained with 10 μ M propidium iodide and imaged with an UltraView ERS spinning-disc confocal microscope (Perkin-Elmer Life and Analytical Sciences, Waltham, MA, USA) equipped with a 40× objective. Propidium iodide was excited using the 561-nm line of the argon-krypton laser and emission was detected at 615 nm. More than 200 optical sections of a root hair were taken at 0.2 μm intervals, and the image data were projected using Volocity software version 6.3 (Improvision).

### Imaging ROP2, RABA4b and a tonoplast marker in living root hairs

For imaging the small GTPases, wild-type plants expressing Enhanced Yellow Fluorescent Protein (EYFP)-ROP2 (Xu and Scheres, 2005) and EYFP-RABA4b (Preuss et al., 2004) were crossed with *ark1-4* plants (Yoo et al., 2008). For imaging vacuolar membrane dynamics, wild-type plants expressing GFP-tonoplast intrinsic protein (TIP; GenBank acc. no: U39485; Cutler et al., 2000) were crossed with *agd1-1 and ark1-4* plants (Yoo et al., 2008). Root hairs were imaged with an UltraView ERS spinning-disc confocal microscope (Perkin-Elmer Life and Analytical Sciences, Waltham, MA, USA) equipped with a ×63 water-immersion objective (Numerical aperture 1.40). EYFP and GFP were excited using the 488-nm line of the argon-krypton laser, and emission was detected at 510 nm. Root hairs were imaged by collecting optical sections at 1 μm intervals. Analyses of EYFP-ROP2 and EYFP-RABA4b localization in growing root hairs were conducted on images of projected stacks of optical sections acquired every 30 s–1 min over a period of 1–2 h. For the analyses of GFP-TIP localization optical sections were acquired every 1 s over a period of 2 min.

## Results

### Enhanced root hair phenotypes in ark1 double mutants

**Figure 1:**
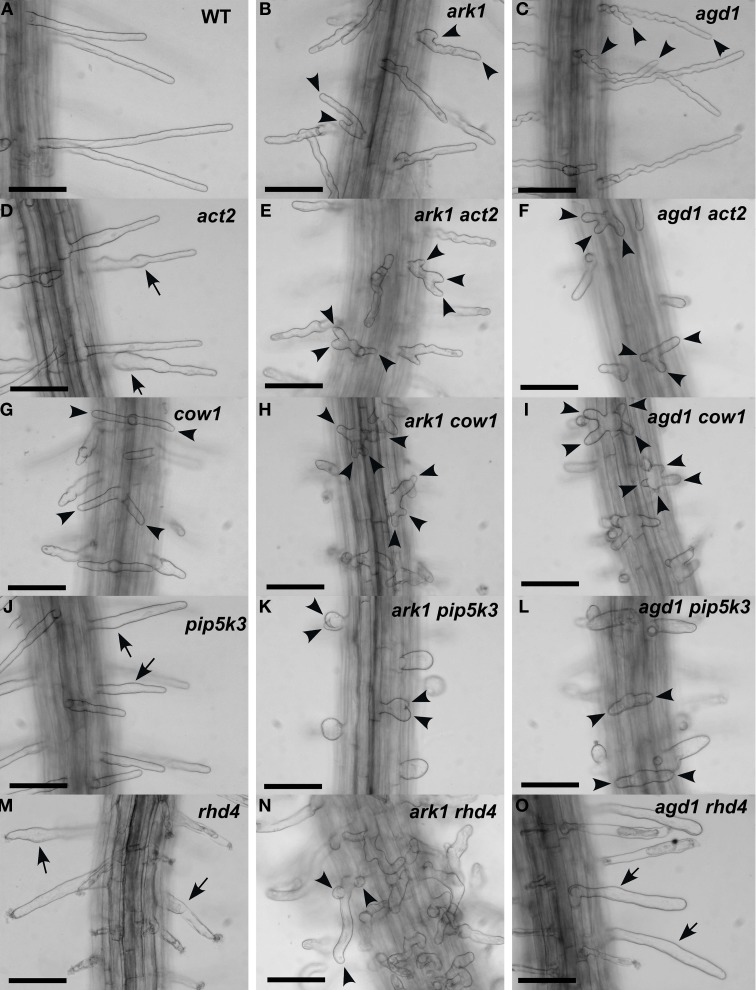
**Root hair phenotypes of *ark1, agd1* and their respective double mutants**. **(A–C)** Wild-type and *ark1*, and *agd1* root hairs. **(D,G,J,M)** Root hairs of single *act2*, *cow1*, *pip5k3*, and *rhd4* mutants. **(E,H,K,N)** Root hairs of double mutants with *ark1*. **(F,I,L,O)** Root hairs of double mutants with *agd1*. Multiple tips from a single root hair initiation point are indicated by arrowheads. Regions of the root hair that swell or bulge are indicated by arrows. Bars = 100 μm.

**Figure 2:**
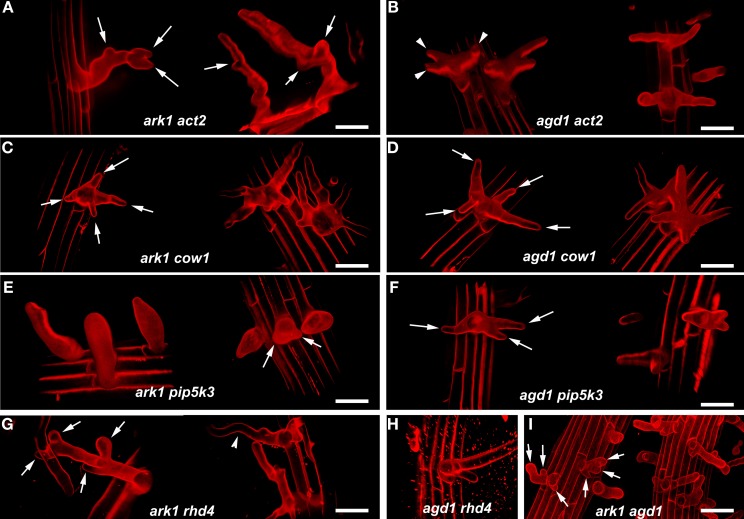
**Representative confocal microscope images of various *ark1* and *agd1* double mutants**. Roots were stained with propidum iodide and a series of optical sections were projected and rendered using Volocity 6.3 software to more accurately visualize the resulting shapes of the double mutants. **(A**,**B)** Note that multiple root hair tips appear to emerge at various points in elongating *ark1 act2* root hairs (arrows) while multiple tips in *agd1 act2* are restricted to early initiation (arrowheads). **(C,D)** In *ark1 cow* and *agd1 cow1*, multiple root hairs tips are prevalent at a single initiation point (arrows). **(E,F)** Bulbous root hairs are a typical feature of *ark1 pip5k3*. Some bulbous root hairs appear to have two tips (arrows). Like *agd1 act2* and *agd1 cow1, agd1 pip5k3* root hairs have multiple root hairs emerging from one initiation point (arrows). **(G)** *ark1 rhd4* root hairs have new tips emerging throughout root hair elongation (arrows) and exhibit wavy growth typical of *ark1* single mutants (arrowhead). **(H)** *agd1 rhd4* root hairs resemble *rhd4* single mutants. **(I)** Root hairs of *ark1 agd1* root hairs are mostly similar to the multiple tip phenotype (arrows) exhibited by *agd1 act2*, *agd1 cow1, ark1 cow1*, and *agd1 pip5k3*. Bars = 50 μm.

**Figure 3:**
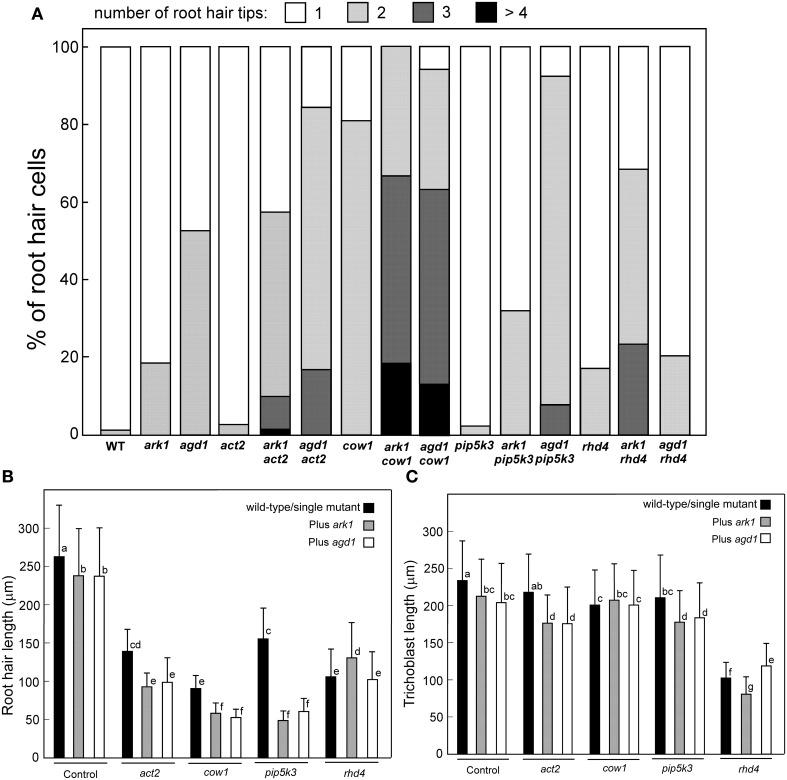
**Quantification of root hair defects in *agd1* and *ark1* single and various mutant combinations**. **(A)** Frequency of multiple tips. Root hair tips from each root hair or initiation site were counted, and numbers of the root hair tips are presented as a percentage of the number of root hairs sampled. More than 100 trichoblasts were sampled for each single mutant/double mutant. Quantification of root hair **(B)** and trichoblast **(C)** length. Black bar indicates the average length of wild-type root hair cells or single *act2, cow1, pip5k3*, and *rhd4* (leftmost black bars). The rightmost gray and unfilled bars represent average root hair cell or trichoblast length of *ark1* and *agd1*, respectively. All other bars show the average root hair length of different double mutant combinations. Data are means (±*SD*) from > 120 root hairs or trichoblasts. Means with different letters are significantly different as determined by Tukey's HSD test (*P* < 0.005).

We compared root hairs of *ark1 act2, ark1 pip5k3, ark1 cow1*, and *ark1 rhd4* with *agd1 act2, agd1 pip5k3, agd1 cow1*, and *agd1 rhd4* using bright field and confocal microscopy (Figures 1, 2). Representative bright field images of wild-type and single *agd1* and *ark1* mutant root hairs are shown in Figures 1A–C while representative bright field images of single *act2, cow1, pip5k3*, and *rhd4* mutants are shown in Figures 1D,G,J,M. Single *act2* mutants, which are disrupted in the gene encoding *ACTIN2*, often had irregular root hair diameters with thicker bases and root hairs that were shorter than wild-type (Ringli et al., 2002; Figure 1D). Quantitative analysis showed that the percentage of *act2* root hairs with two tips and was similar to wild-type and significantly less than *ark1* and *agd1* single mutants (Figure 3A). The average root hair length of *act2* was significantly less than wild-type, and *ark1* and *agd1* single mutants (Figure 3B). Root hairs of *ark1 act2* double mutants showed additive morphological defects (i.e., double mutants had shorter root hairs than their respective single mutant and displayed the wavy and branched phenotypes characteristic of single *ark1* mutants; compare Figures 1E with 1B; Figure 3B). However, unlike *agd1 act2*, which often had two or three tips restricted to the initiation site (Figures 1F, 2B), *ark1 act2* produced additional tips not only at initiation but also during the root hair elongation stage (Figures 1E, 2A).

The *COW1*mutant, which is disrupted in the gene encoding a sec14p domain phosphatidyl inositol (PtdIns) transfer protein (PITP) (Böhme et al., 2004; Vincent et al., 2005) was previously identified as an enhancer of *agd1*(Yoo et al., 2012). Single mutants of *cow1* typically had two tips originating from one initiation site (Figures 1G, 3A), while *agd1 cow1* had root hairs with up to five tips originating from one initiation site (Figures 1I, 2D, 3A). Double mutants of *ark1 cow1* also had a large percentage of root hairs with up to four to five tips (Figures 1H, 2C, 3A) indicating that *cow1* enhances *ark1* defects similar to *agd1*. Root hairs of *ark1 cow1* and *agd1 cow1* were significantly shorter than root hairs of their respective single mutants (Figure 3B).

*PIP5K3* encodes a type B phosphatidylinositol-4-phosphate 5-kinase 3 that catalyzes the formation of phosphatidylinositol 4,5-bisphosphate (PI-4,5P2) from PI-4P. Consistent with previous studies, a mutation in *PIP5K3* resulted in plants with root hairs that were shorter and slightly thicker than wild-type (Kusano et al., 2008; Stenzel et al., 2008; Figures 1J, 3B). The *ark1 pip5k3* double mutants had stunted root hair growth manifested visually by the formation of short bulbous structures. Although a majority of root hairs of *ark1 pip5k3* formed bulbous structures, some root hairs displayed rudiments of what appeared to be two tips (Figures 1K, 2E). On the other hand, the multiple tips that formed in root hairs of *agd1 pip5k3* double mutants were very distinct and quantitative analysis showed that these were more numerous than *ark1 pip5k3* (Figures 1L, 2F, 3A). The root hair length of the double mutants of *pip5k3* to either *ark1* or *agd1* was synergistically reduced (Figures 1J–L, 3B). Although overall reduction in root hair length was similar between *ark1 pip5k3* and *agd1 pip5k3* (Figure 3B), the overall shape of *ark1 pip5k3* was clearly different from that of *agd1 pip5k3* (compare Figures 1K,L and Figures 2E,F).

As reported previously, *RHD4* encodes a PI-4P phosphatase. Root hairs of *rhd4* mutants were short, branched and randomly formed bulges along their length (Thole et al., 2008; Figure 1M). Previously, we found that *agd1 rhd4* root hairs showed similar root hair phenotypes as the single *rhd4* mutant suggesting that *rhd4* is epistatic to *agd1* (Figures 1M,O, 2H; Yoo et al., 2012). Here we found that the *ark1 rhd4* double mutant exhibited additive effects. For example, *ark1 rhd4* had swollen root hairs that bulged in random positions, which was a typical phenotype of *rhd4* single mutants. Root hairs of *ark1 rhd4* also showed curling and branching along their length, a feature characteristic of *ark1* single mutants (Figures 1B,M,N, 2G). Quantitative analysis of the percentage of root hairs with multiple tips and average root hair length confirmed that *rhd4* is epistatic to *agd1* but not to *ark1* (Figures 3A,B). In agreement with our previous report, *ark1 agd1* root hairs were shorter than their respective single mutants and exhibited multiple tips from one initiation point (Figure 2I).

We also measured trichoblast length in the various single and double mutant combinations. Based on this analysis, we found that *agd1* and *ark1* had slight but statistically significant reduction in trichoblast length compared to wild-type (Figure 3C). The reduction in trichoblast length was enhanced in *ark1 act2*, *agd1 act2*, *ark1 pip5k3*, and *agd1 pip5k3* double mutants but not in *ark1 cow1* and *agd1 cow1*. It was also found that *rhd4* single mutants had dramatically reduced trichoblast length compared to wild-type and all other single mutants examined. However, when combined with a mutation in the *ARK1* or *AGD1* gene, opposite effects on trichoblast length were observed. It was found that *ark1 rhd4* had shorter trichoblasts than *rhd4* while those on *agd1 rhd4* were longer (Figure 3C). No differences in the location of root hairs along the trichoblasts were observed.

We then measured primary root length of single and double mutants under conditions used for examining root hair and trichoblast length to determine if differences in trichoblast length correlated with altered primary root length. We found that the shorter trichoblast in *rhd4*, *ark1 rhd4*, and *agd1 rhd4* translated into shorter primary roots compared to all other single or double mutant combinations. However, the longer trichoblasts in *agd1 rhd4* compared to *rhd4* or *ark1 rhd4* were not correlated with longer primary roots. Interestingly, we also found that *cow1* primary roots were shorter than wild-type but was restored to wild-type lengths in *ark1 cow1* (Figure S1).

### RABA4b- and ROP2-GTPase targeting to the root hair tip is disrupted in ark1

**Figure 4:**
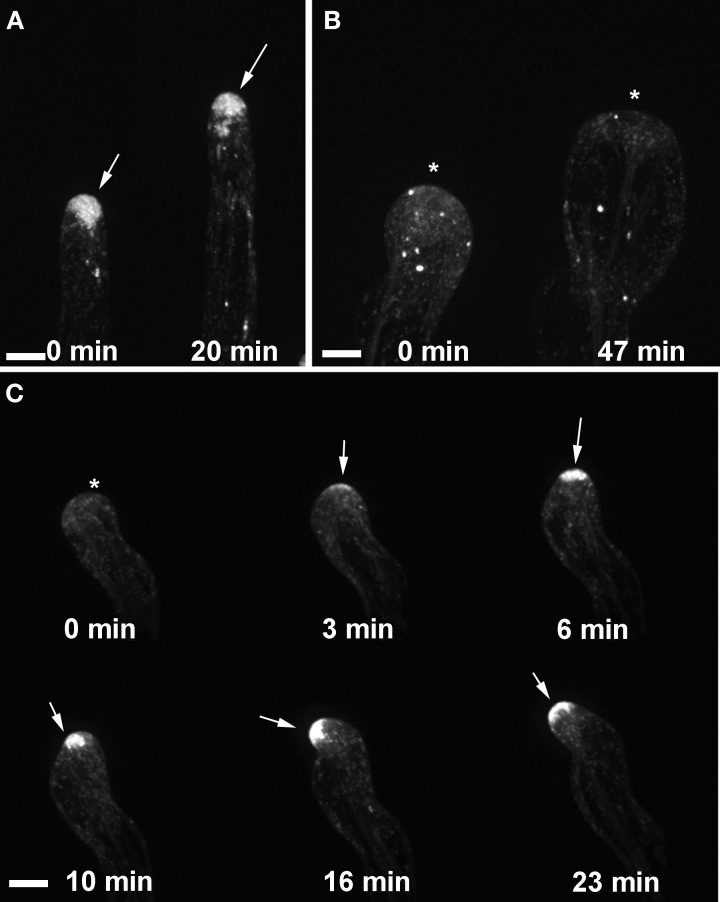
**Targeting of RABA4b secretory vesicles to the root hair tip is disrupted in *ark1***. **(A)** EYFP-RABA4b is preferentially enriched at the tips of wild-type seedlings during root hair elongation (arrows). **(B)** In *ark1* root hairs, there is no enrichment of the EYFP-RABA4b signal in a swelling root hair (asterisks). **(C)** In a waving root hair, the EYFP-RABA4b signal often dissipates (asterisk) but eventually reforms when tip growth resumes (arrows). The EYFP-RABA4b typically follows the change in root hair growth direction. Time-lapse movie sequences of corresponding still images are presented as Movies S1 (for wild-type), S2, and S3 (for *ark1*). Bars = 20 μm.

We showed previously that the wavy root hair growth of *agd1* was accompanied by mistargeting of tip-localized RabA4b trans-Golgi compartments (Yoo et al., 2012). Given the similar root hair defects of *agd1* and *ark1*, we asked whether root hairs of *ark1* also show aberrant RabA4b dynamics. Using spinning disc confocal microscopy, we found that EYFP-RabA4b signal often dissipated as the root hair elongated in contrast to the consistent signal observed in wild-type (Figures 4A–C; Movies S1–S3). Furthermore, swelling root hairs of *ark1* did not show any preferential EYFP-RabA4b accumulation (Figure 4B). As shown in Movie S2, EYFP-RabA4b accumulated at the root hair apex of swelling *ark1* root hairs but the signal would quickly dissipate. Like in the previously reported *agd1* root hairs, EYFP-RabA4b signal in waving root hairs of *ark1* constantly shifted at the growing apex such that the relocalization of the signal was followed by a change in the direction of root hair growth (Figure 4C; Movie S3).

**Figure 5:**
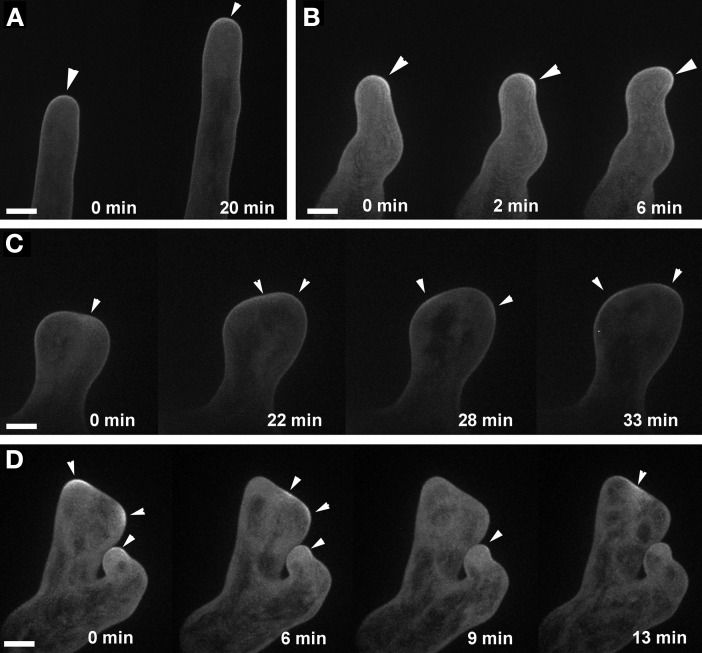
**ROP2 localization to the apical cell membrane of root hairs is altered in *ark1*. (A)** A wild-type root hair shows enhancement of EYFP-ROP2 at the apical plasma membrane (arrow heads). **(B)** In a wavy root hair of *ark1*, plasma membrane localized EYFP-ROP2 shifts position prior to the change in root hair growth direction (arrowheads). **(C,D)** In *ark1* root hairs that swell and form branches, EYFP-ROP2 signal appears to become enriched at more than one location within the cell. Also, the EYFP-ROP2 signal dissipates then shifts to a different plasma membrane domain (arrowheads). Time-lapse movie sequences of still images in panels **(A**,**C)** are presented as Movies S4 (for wild-type) and S5 (for *ark1*), respectively. Bars = 20 μm.

Targeting of plasma-membrane ROP2 was altered in *ark1* root hairs similar to what was observed in *agd1* (Yoo et al., 2012). In root hairs of *ark1*, EYFP-ROP2 localized to the apical plasma membrane (Figure 5A). However, unlike wild-type root hairs, the EYFP-ROP2 signal in wavy root hairs of *ark1* shifted to the side where tip growth changed direction (Figure 5B). In *ark1* root hairs that showed extreme polarity defects, intense plasma-membrane EYFP-ROP2 signal would alternately shift from one defined site to another. As such, the root hair was never able to attain the tubular shape typical of wild-type root hairs (Figures 5C,D; Movies S4, S5).

### Vacuolar membrane dynamics is altered in tips of agd1 and ark1 root hairs

**Figure 6:**
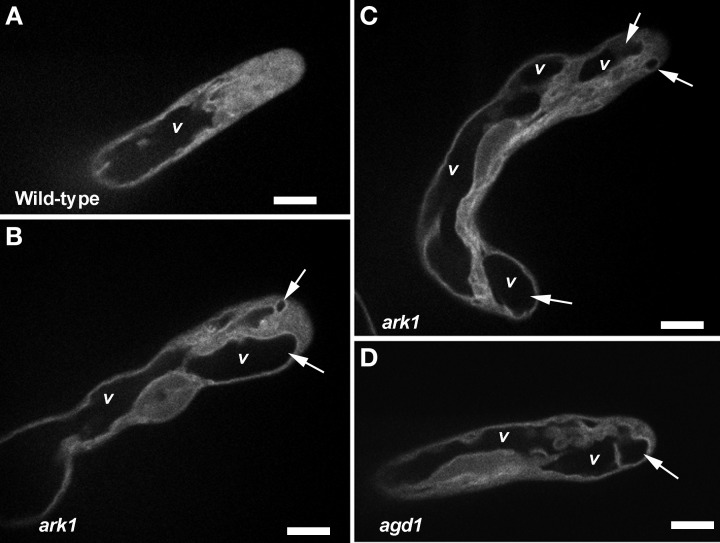
**Abnormal tonoplast organization at the growing apex was observed in *ark1* and *agd1* root hair cells**. Tonoplast was visualized by expressing GFP-TIP in the plants. **(A)** In wild-type, the vacuole (*v*) is maintained at a distance from the extreme root hair apex. **(B–D)** In the *ark1* and *agd1* root hairs the vacuolar membranes occasionally protrude into the extreme apex (arrows). Time-lapse movie sequences of corresponding still images are presented as Movies S6 (for wild-type), S7 (*ark1*), and S8 (for *agd1*). Bars = 20 μm.

We next sought to determine whether *agd1* and *ark1* root hairs exhibited other defects in membrane organization that could be linked to cellular tip abnormalities reported previously (Yoo et al., 2008, 2012). We looked closely at the dynamics of the vacuolar membrane because of its dependence on the organization of actin and microtubules (Higaki et al., 2006; Oda et al., 2009), which are bundled in tips of *agd1* and *ark1* root hairs (Yoo et al., 2008). To image the vacuolar membrane, we expressed GFP-TIP in *agd1* and *ark1* (Cutler et al., 2000). In actively elongating wild type root hairs, GFP-TIP was highly dynamic and the membrane delineating the tonoplast was restricted to a subapical region of the root hair (Figure 6A; Movie S6). On the other hand, GFP-TIP delineated vacuolar membranes occasionally protruded into the extreme apex (Figures 6B–D; Movies S7, S8).

## Discussion

Previously, we reported that *agd1* and *ark1* had similar root hair and cytoskeletal defects. This suggested that despite predicted differences in the functions of the AGD1 and ARK1 proteins, they likely share common molecular targets in defining root hair growth directionality and polarity (Yoo et al., 2008). It was shown, however, that the vesicle trafficking inhibitor, BFA, completely rescued *agd1* root hair defects but not those of *ark1*. Furthermore, analysis of the root hair phenotypes of double *ark1 agd1* mutants revealed that *agd1* was not epistatic to *ark1* (Figure 2I; Yoo et al., 2008). Taken together these results indicate that the pathways where AGD1 and ARK1 function may diverge at certain points along the root hair developmental program (Yoo et al., 2008). To further tease apart the stages of root hair development where ARK1 and AGD1 might share common molecular targets and where they might diverge, we conducted genetic interaction studies and live cell imaging of root hair polarity markers in the *ark1* mutant similar to previous studies with *agd1* (Yoo et al., 2012).

The resulting root hair phenotypes of various *ark1* double mutants and how they compared with the *agd1* double mutants reported previously (Yoo et al., 2012) provided some clues as to points in the root hair developmental network where *ARK1* might diverge from *AGD1*. For example, the predominant morphological defect of *ark1 act2* double mutants was the formation of branches/multiple tips along the entire length of their root hairs. In contrast, *agd1 act2* defects were expressed primarily as the formation of multiple tips at the initiation site (Figures 1, 2; Yoo et al., 2012). These results indicate that ARK1 in conjunction with actin might be involved in the maintenance of polarity throughout root hair development (i.e., from root hair initiation to tip growth). On the other hand, AGD1 is likely to exert its predominant effects on actin-dependent root hair developmental processes during root hair initiation. The notion that *ARK1* might have a broader function in root hair development than *AGD1* is further supported by the observation that like *ark1 act2* double mutants, *ark1 rhd4* root hairs had the tendency to produce an excessive number of tips and bulges that formed along the entire root hair as it elongated. With regard to how *ARK1* might function in concert with *COW1*, the multiple initiation sites of the *ark1 cow1* double mutant were very similar to those observed in *agd1 cow1*. This indicates that ARK1 and AGD1 converge on the Sec14p-like phosphatidylinositol transfer protein encoded by *COW1* with such interaction impacting mostly the events that occur during root hair initiation.

It is noteworthy that double mutants of *ark1 rhd4* had longer root hairs than *rhd4* single mutants (Figure 3B). The longer root hairs could be explained by the obvious absence of ruptured root hairs in *ark1 rhd4* compared to *rhd4* single mutants (Figures 1M,N). Earlier reports showed that *rhd4* over-accumulated PI-4P on internal membrane compartments rather than the plasma membrane (Thole et al., 2008), which might contribute to increased delivery of cell wall materials to the tip resulting in bulging and bursting of the root hairs (Galway et al., 1999). *ARK1* could be functioning as a suppressor of *RHD4* in regard to root hair length by redirecting vesicles containing PI-4P that typically over-accumulate in *rhd4* mutants to the newly developing tips of *ark1 rhd4*. As a result excess PI-4P is diluted or retargeted to their correct location on the plasma membrane, preventing premature root hair rupture. It would be interesting to see using PI-4P biosensors (Vermeer et al., 2009) whether the dynamics of PI-4P in *ark1 rhd4* differs from that of *rhd4*. Interestingly, whereas *ARK1* appears to be a suppressor of *RHD4* with regard to root hair length, a lesion in *AGD1* seems to suppress the shorter trichoblast length resulting from a mutation in *RHD4* (Figure 3C). Although the mechanisms on how this is accomplished are unknown, our data point to another level of divergence between ARK1 and AGD1 where AGD1 might function in both RHD4-mediated tip and diffuse growth processes.

Whereas AGD1 contains a pleckstrin homology (PH) domain that binds to PIs (Vernoud et al., 2003; Yoo et al., 2012), ARK1 has not been biochemically characterized for PI interactions. However, in a recent study of neuronal axons, which partly mirror root hair tip growth, it was reported that PIPKα is not only involved in PI metabolism but directly promotes microtubule depolymerizing activity of the kinesin, KIF2A (Noda et al., 2012). Although the mechanisms by which ARK1 regulates microtubule organization remain to be elucidated (Zhu and Dixit, 2012), it is tempting to speculate that its activity might also be influenced by components of PI metabolism similar to what has been demonstrated for neurite development. It is possible that actin binding to the armadillo repeat-containing domain of ARK1 (Yang et al., 2007) might somehow be linked to ARK1 crosstalk with PIs. As noted earlier, the similar root hair phenotypes of *ark1 cow1* and *agd1 cow1* indicate that such cross-talk might be accomplished via the PI transfer protein, COW1.

Similar patterns of RabA4B and ROP2 mistargeting in *ark1* root hairs to that shown for *agd1* were observed (Figures 4, 5; Movies S2, S3, S5; Yoo et al., 2012). Such defects could be attributed to common cytoskeletal defects observed in both mutants (Yoo et al., 2008). Furthermore, we found that both *ark1* and *agd1* root hairs mirrored each other in terms of abnormalities in vacuolar membrane organization. It has been reported that the cytoskeleton is important in regulating plant vacuolar structure and dynamics (Higaki et al., 2006; Oda et al., 2009). The spatially close localization of F-actin and vacuolar membrane, and the movement of F-actin along cytoplasmic strands adjacent to large vacuoles, is likely responsible for actin-dependent regulation of vacuolar dynamics. Moreover, it has been demonstrated that actin or microtubule inhibitors induced smaller vacuolar compartments that detached from the larger vacuoles (Higaki et al., 2006; Oda et al., 2009). In *ark1* and *agd1* mutants, the vacuolar membranes continuously formed small compartments at the tip (Movies S7, S8) suggesting that the disruption in their cytoskeleton parallels that induced by cytoskeletal inhibitors. Taken together, our live cell imaging studies identify additional downstream molecular targets common to ARK1 and AGD1 in the maintenance of root hair polarity.

**Figure 7:**
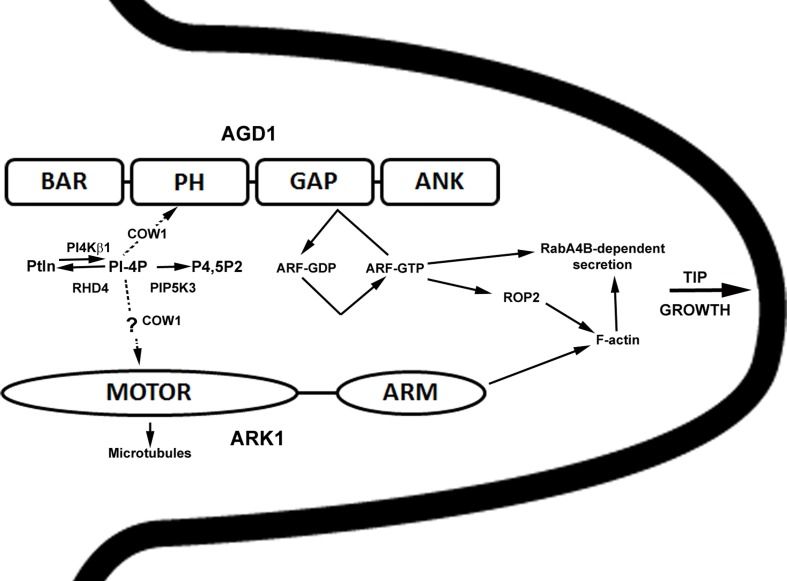
**Simplified and speculative model for ARK1- and AGD1- mediated control of root hair tip growth**. The various domains of AGD1 and ARK1 proteins are shown. Bin1-Amphiphysin-Rvs167p/Rvs161p (BAR), Pleckstrin Homology (PH), GAP, and Ankyrin repeat (ANK) for AGD1 (Vernoud et al., 2003) and kinesin MOTOR and armadillo repeat-containing (ARM) domains for ARK1 (Yang et al., 2007). In this model, RHD4, PI4Kβ1, and PIP5K3 influence AGD1 activity by their timely depletion and synthesis of PI monophosphates (e.g., PI-4P; Preuss et al., 2006; Kusano et al., 2008; Stenzel et al., 2008; Thole et al., 2008). COW1 is involved in the transfer of PIs (dashed arrow; Phillips et al., 2006) to facilitate binding to PH domain of AGD1 (Yoo et al., 2012) or possible interaction with ARK1. PI binding to AGD1 would in turn modify the activity of a yet to be determined ARF-GTPase. Because ARF-GTPase has been shown to control ROP2 targeting (Xu and Scheres, 2005), AGD1 could influence F-actin organization indirectly and as a result mediate RabA4B and vacuolar dynamics (Preuss et al., 2004; Yoo et al., 2008, 2012). AGD1 and ARK1 could act in parallel pathways where ARK1 mediates crosstalk between microtubules (via the kinesin motor domain) and F-actin (via the ARM domain) (Yang et al., 2007). Although genetic interaction studies presented here implicate PIs in the ARK1 function, possibly through COW1 (dashed arrow), the precise mechanisms by which this is accomplished is unclear.

In summary, our studies revealed that ARK1, an unconventional plant kinesin, is an important component that ties membrane organization to the cytoskeleton during root hair development. Genetic interaction and cell biological data presented here continue to point to an interaction between ARK1 and AGD1 in molecular pathways that modulate tip growth, and such cross-talk occurs in defined steps within the root hair developmental program that involve PI metabolism. A proposed model speculating on how ARK1 and AGD1 might function in root hair development is presented in Figure 7. Whereas AGD1 exerts its greatest impact at the early stages of root hair initiation and tip growth, ARK1 has a broader role that covers the entire root hair developmental program.

## Author contributions

Cheol-Min Yoo generated double mutants and mutant plants expressing membrane polarity and tonoplast markers. Elison B. Blancaflor conducted live cell imaging of *ark1* root hairs. Cheol-Min Yoo and Elison B. Blancaflor analyzed the data and wrote the paper.

### Conflict of interest statement

The authors declare that the research was conducted in the absence of any commercial or financial relationships that could be construed as a potential conflict of interest.

## Supplementary material

Figure S1 **Primary root length of various single and double mutants.** Data are means (±*SD*) from primary roots of 24 seedlings. Means with different letters are significantly different as determined by Tukey's HSD test (*P* < 0.005).

Movie S1 **EYFP-RABA4b localization in an elongating wild-type root hair.** Total elapsed time of the movie is 1 h.

Movie S2 **EYFP-RABA4b localization in a swelling *ark1* root hair.** Total elapsed time of the movie is 1 h.

Movie S3 **EYFP-RABA4b localization in an *ark1* root hair that constantly shifts direction.** Total elapsed time of the movie is 1 h.

Movie S4 **EYFP-ROP2 localization in an elongating wild-type root hair.** Total elapsed time of the movie is 1 h.

Movie S5 **EYFP-ROP2 localization in a swelling *ark1* root hair.** Total elapsed time of movie is 1 h.

Movie S6 **Vacuolar membrane dynamics visualized by GFP-TIP expression in a wild-type root hair.** Total elapsed time of movie is 2 min.

Movie S7 **Vacuolar membrane dynamics visualized by GFP-TIP expression in an *ark1* root hair.** Total elapsed time of movie is 2 min.

Movie S8 **Vacuolar membrane dynamics visualized by GFP-TIP expression in an *agd1* root hair.** Total elapsed time of movie is 2 min.
