# Supplementary figures and images for: Overlapping and divergent signaling pathways for ARK1 and AGD1 in the control of root hair polarity in Arabidopsis thaliana
Source: Front Plant Sci. 2013 Dec 24;4:528. doi: 10.3389/fpls.2013.00528 (PMC3871054; doi:10.3389/fpls.2013.00528)

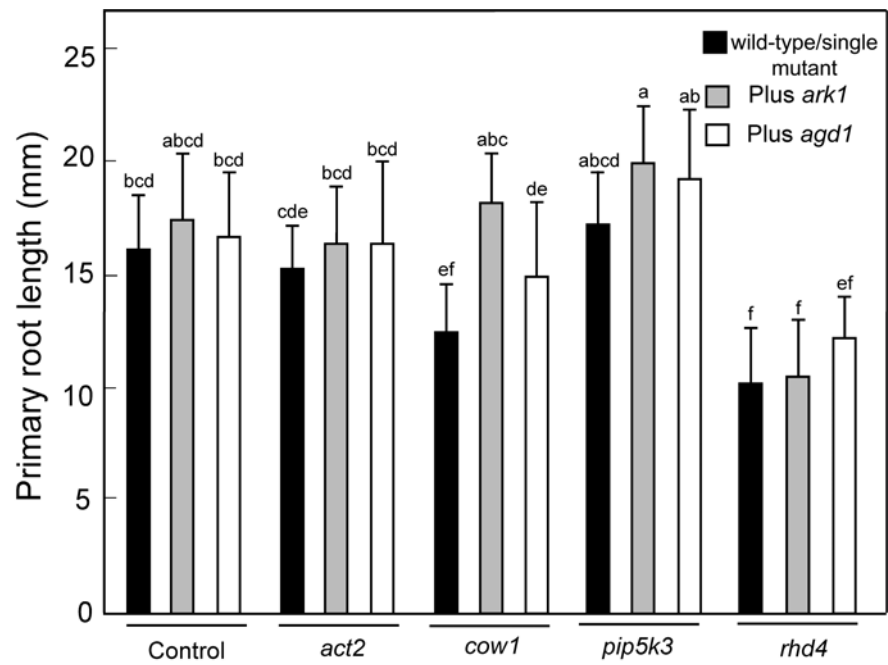

Supplement: Figure S1 — Primary root length of various single and double mutants. Data are means (±SD) from primary roots of 24 seedlings. Means with different letters are significantly different as determined by Tukey's HSD test (P < 0.005). [file DataSheet1.PDF]
